# Assembly and Genome Annotation of Different Strains of Apple Fruit Moth Virus (*Cydia pomonella* granulovirus)

**DOI:** 10.3390/ijms25137146

**Published:** 2024-06-28

**Authors:** Tatiana N. Lakhova, Aleksandra A. Tsygichko, Alexandra I. Klimenko, Vladimir Y. Ismailov, Gennady V. Vasiliev, Anzhela M. Asaturova, Sergey A. Lashin

**Affiliations:** 1 Kurchatov Genomic Centre of Institute of Cytology and Genetics SB RAS, 630090 Novosibirsk, Russia; klimenko@bionet.nsc.ru (A.I.K.); lashin@bionet.nsc.ru (S.A.L.); 2 Department of Mathematics and Mechanics, Mathematical Center, Novosibirsk State University, 630090 Novosibirsk, Russia; 3 Federal State Budgetary Scientific Institution, Federal Research Center of Biological Plant Protection, 350039 Krasnodar, Russia; 23612361@inbox.ru (A.A.T.); biocontrol-vniibzr@yandex.ru (A.M.A.); 4 Faculty of Natural Sciences, Novosibirsk State University, 630090 Novosibirsk, Russia

**Keywords:** *Cydia pomonella* granulovirus, codling moth, genome sequencing, comparative genomic analysis

## Abstract

*Cydia pomonella* granulovirus is a natural pathogen for *Cydia pomonella* that is used as a biocontrol agent of insect populations. The study of granulovirus virulence is of particular interest since the development of resistance in natural populations of *C. pomonella* has been observed during the long-term use of the Mexican isolate CpGV. In our study, we present the genomes of 18 CpGV strains endemic to southern Russia and from Kazakhstan, as well as a strain included in the commercial preparation “Madex Twin”, which were sequenced and analyzed. We performed comparative genomic analysis using several tools. From comparisons at the level of genes and protein products that are involved in the infection process of virosis, synonymous and missense substitution variants have been identified. The average nucleotide identity has demonstrated a high similarity with other granulovirus genomes of different geographic origins. Whole-genome alignment of the 18 genomes relative to the reference revealed regions of low similarity. Analysis of gene repertoire variation has shown that BZR GV 4, BZR GV 6, and BZR GV L-7 strains have been the closest in gene content to the commercial “Madex Twin” strain. We have confirmed two deletions using read depth coverage data in regions lacking genes shown by homology analysis for granuloviruses BZR GV L-4 and BZR GV L-6; however, they are not related to the known genes causing viral pathogenicity. Thus, we have isolated novel CpGV strains and analyzed their potential as strains producing highly effective bioinsecticides against *C. pomonella*.

## 1. Introduction

Biocontrol methods are an alternative to the use of synthetic pesticides against crop insect pests. These methods include the use of compounds of biological origin, predators, competitors, and pathogens (bacteria, fungi, and baculoviruses) with entomopathogenic properties [1,2]. Biological control is a key component of the “systems approach”, which not only reduces the population of phytophages but also does not disrupt natural biocoenotic relationships in populations [3].

Among biocontrol agents, *Cydia pomonella* granulosis virus (CpGV) from the Baculoviridae family is considered to be one of the most effective against the target object, with an efficiency of 95–97% [4]. On its basis, there are commercial preparations named “CYD-X”, “Madex Pro”, “Madex Twin”, “Madex Top”, “Carpovirus”, and “Carpovirus Plus” [5,6]. The main properties of CpGV, in addition to high efficacy, are selectivity action, ability to infiltrate into pest populations, and safety for the environment [7].

Since the target insect for CpGV is *Cydia pomonella*, bioinsecticides based on CpGV are mainly used to protect apple orchards. The area of apple orchards in Europe is about 470 thousand hectares. The major apple-producing countries are Poland, Spain, and France [8,9]. The area of apple orchards in the Russian Federation is about 220 thousand hectares. The south of Russia is a leader in apple production (Kabardino-Balkaria, Stavropol Krai, Volgograd Oblast, and Krasnodar Krai) [10,11,12].

It should be noted that the Mexican isolate, which was one of the first to be isolated, is the most common and serves as the basis for many commercial preparations. Its genome was sequenced and fully assembled into a ring chromosome in 2001 [13]. Nevertheless, in the process of its long-term use, the formation of resistance in natural populations of *C. pomonella* has been noted [14]. Researchers distinguish three types of resistance to baculovirus depending on inheritance. Type I resistance is determined by the sex Z chromosome, type II by the predominant autosome, and type III is both autosomal and Z-linked [14,15,16,17]. Thus, this indicates a necessity for the constant search and use of new highly effective strains that can overcome the acquired resistance of *C. pomonella*. That is why it is expedient to isolate and study the properties of new CpGV strains.

To date, several complete assemblies of CpGV genomes from China, Iraq, South Africa, etc., as well as fragmented genomes of varying degrees of completeness, can be found in open sources; however, no data on CpGV viruses from Russia were found in open sources. On the basis of the Federal State Budgetary Scientific Institution’s “Federal Scientific Center for Biological Plant Protection”, a bioresource collection called the “State Collection of Entomoacariphages and Microorganisms” contains a number of CpGV strains with entomopathogenic properties.

The aim of this study is to perform molecular–genetic identification and describe the genome properties of new CpGV strains indigenous to the south of Russia and Kazakhstan using the methods of whole-genome sequencing and comparative genomic analysis for the first time in the Russian Federation. Thus, the assembly and annotation of genomes of new CpGV strains native to southern Russia will allow us to expand the range of promising entomopathogenic agents against *C. pomonella*, which may become the basis for highly effective bioinsecticides in the future.

## 2. Results

### 2.1. Preparation of Samples for Sequencing and Quality Analysis of Libraries of Entomopathogenic Virus Strains

During library preparation, isolation of a DNA fraction of more than 150 bp allowed to cut off impurities of highly degraded host DNA and degraded viral DNA.

It was found that the fraction of 150–400 bp is 4.5–10% of the total DNA, while 50–80% indicated either a high level of virus degradation during prolonged storage as a suspension or a noticeable contamination of the material with degraded host DNA adhering to the surface of viral particles. The concentrations of starting DNA, prepared libraries, their molarities, and individual barcode identifiers are given in the Supplementary Materials (Table S1).

The obtained libraries of 18 genomes of CpGV from the bioresource collection of the Federal Research Center of Biological Plant Protection’s “State Collection of Entomoacariphages and Microorganisms” and the genome of the strain-producing bioinsecticide “Madex Twin” were sequenced by paired-end reads of 2 × 150 bp. The real volumes of the obtained data after quality filtering amounted to more than 20 million paired-end reads for each strain.

### 2.2. Preparing and Filtering Sequenced Data

Raw data underwent quality control and trimming was performed, followed by mapping to the CpGV reference genome (NC_002816.1). Then, statistics were calculated from the mapped reads to the reference (NC_002816.1). The characteristics are presented in the Supplementary Materials (Table S2). Coverage rates were calculated using the Lander–Waterman formula [18], L*N/G, where L is the number of paired-end reads mapped to the reference genome, N is the length of the reads, and G is the length of the genome.

### 2.3. CpGV Genome Assembly

Two assemblers were selected to acquire draft assemblies: Spades [19,20] and MinYS [21]. When selecting mapped reads and translating them into fastq files for subsequent assembly, files with paired-end reads and files with single reads that had not had a pair mapped to the genome were obtained. Since MinYS does not handle single reads, paired-end reads were taken before mapping to the reference genome. In this case, the assembler independently mapped the reads to the reference genome.

The Pilon tool was applied to each assembly to reduce possible assembly errors [22] and acquired the corrected assembly. After this step, the quality of the assemblies was also evaluated using Quast tool [23] in order to track for each genome whether the parameters had degraded or not. Then, using the Gfinisher tool [24], we obtained a more complete assembly based on the two existing draft assemblies. The main assembly was Spades and the additional one was MinYS because the Spades one had better assembly quality metrics. Gfinisher reduced the number of resulting contigs in almost all assemblies.

Finally, 18 assemblies were obtained, each of which consisted of 4 contigs on average. The main characteristics of the final assemblies of CpGV genomes—obtained as a result of improving the draft assemblies of Spades and MinYS using Pilon and Gfinisher—are summarized in Table 1.

### 2.4. Genome Annotation

Genome annotation was performed using the Prokka tool [25]. Table 2 summarizes the genomic annotation of the virus compared to the reference genome NC_002816.1. 

Genome assemblies and annotations are deposited at NCBI. Accession numbers of the sequences at NCBI are listed in the Supplementary Materials (Table S3).

#### Further Annotation and Analysis of Genes and Their Products Involved in the Virosis Infectious Process

When studying genetic variability in different strains of apple moth granulovirus, the genes involved in the infectious process of *C. pomonella* virosis are of key importance since the differences between them observed in the studied strains, in comparison with each other and the CpGV reference genome may be the cause of changes in the virulence indices of the corresponding strains. The protein products of the corresponding genes and their associated functions are presented in Table 3.

Genes encoding Chitinase and Cathepsin were automatically annotated by Prokka. Pairwise alignment revealed the sequences of genes encoding IAP and MMP in the studied CpGV genomes (identity = from 99.5 to 100%, e-value = 0.0, alignment length of protein (%) = 100). 

**Figure 1:**
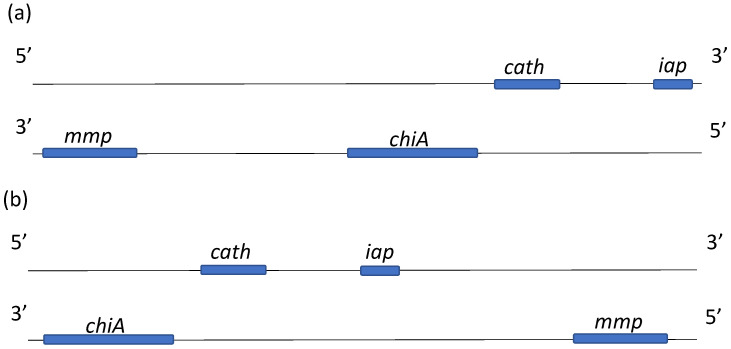
Schematic arrangement of genes involved in the infection process in *C. pomonella* in the CpGV genome: (**a**) the *mmp* gene is located downstream relative to the *chiA* gene on the lagging DNA strand; (**b**) the *mmp* gene is located upstream relative to the *chiA* gene on the lagging DNA strand.

Structurally, the *mmp* gene encoding the MMP protein in the studied genomes can be located in two locations relative to the genes encoding the Chitinase, Cathepsin, and IAP proteins (for those variants where all genes are on the same contig). The first case is observed in most of the genomes of the studied strains (BZR GV: 1, 3, 4, 7–9, 13, L-2, and L-7): the *mmp* gene is located downstream of the *chiA* gene encoding the Chitinase protein (Figure 1a). In the second case, the *mmp* gene is upstream of the *chiA* gene (Figure 1b). This variant is found in the BZR GV genomes: L-4, L-6, and L-8. In the reference genome, these genes are located as depicted in Figure 1b.

**Figure 2:**
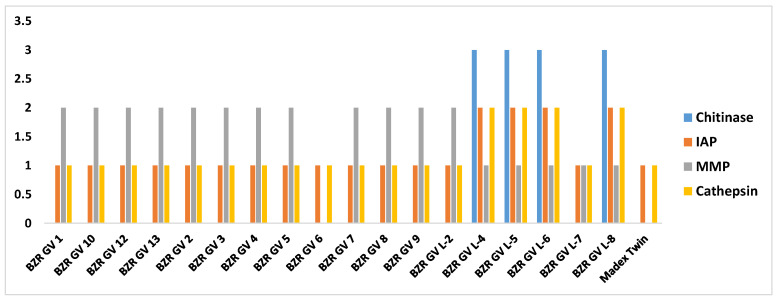
Distribution of the total number of nonsynonymous substitutions in proteins for each CpGV strain, where X-axis shows virus strains, Y-axis shows the number of amino acid substitutions in the corresponding protein. Color-coding by proteins: blue—Chitinase, orange—IAP, gray—MMP, and yellow—Cathepsin.

MAFFT multiple alignment [32] has revealed mutations in genes—specific substitutions are listed in the Supplementary Materials (Table S4), some of them were synonymous (did not result in an amino acid substitution) and nonsynonymous (resulted in an amino acid substitution). Figure 2 shows the distribution of nonsynonymous substitutions found in proteins relative to each CpGV genome examined.

### 2.5. Comparative Genomic Analysis

The next step was to perform comparative genomic analysis to identify and evaluate differences in the genomes of the studied CpGV strains from those represented in NCBI Virus. First, we performed average nucleotide identity (ANI) analysis to establish the degree of closeness between strains based on the study of whole-genome homology. Then, whole-genome alignment of the studied genomes to a reference (NC_002816.1) was performed to search for genomic rearrangements. We also analyzed the variation in gene repertoire relative to the two genomes (NC_002816.1 and KM217575).

#### 2.5.1. ANI Analysis

**Figure 3:**
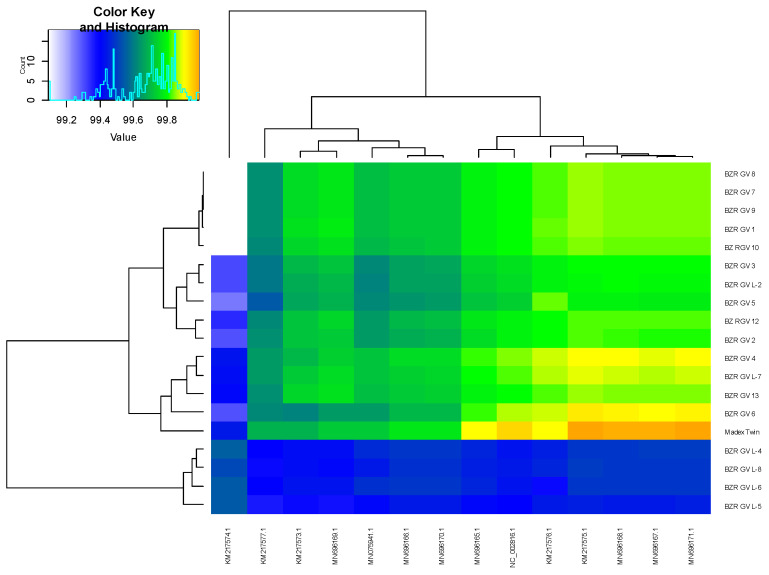
The heatmap of similarity between genomes based on fastANI result, where genome identifiers from NCBI Virus database are marked at the bottom, and identifiers of the studied genomes are marked at the right side. The color scale is set: light blue to blue—(99, 99.4], blue to green—(99.4, 99.8], green to yellow—(99.8, 99.90] and yellow to orange—(99.90, 100]. These interval values were chosen based on the ANI values obtained.

The average nucleotide identity analysis [33] yielded ANI values > 99% for all 18 genomes compared to 14 genomes from NCBI (Figure 3). The obtained values indicate a good proximity of the studied genomes to the genomes represented in the database.

#### 2.5.2. Whole-Genome Alignment of Genomes

**Figure 4:**
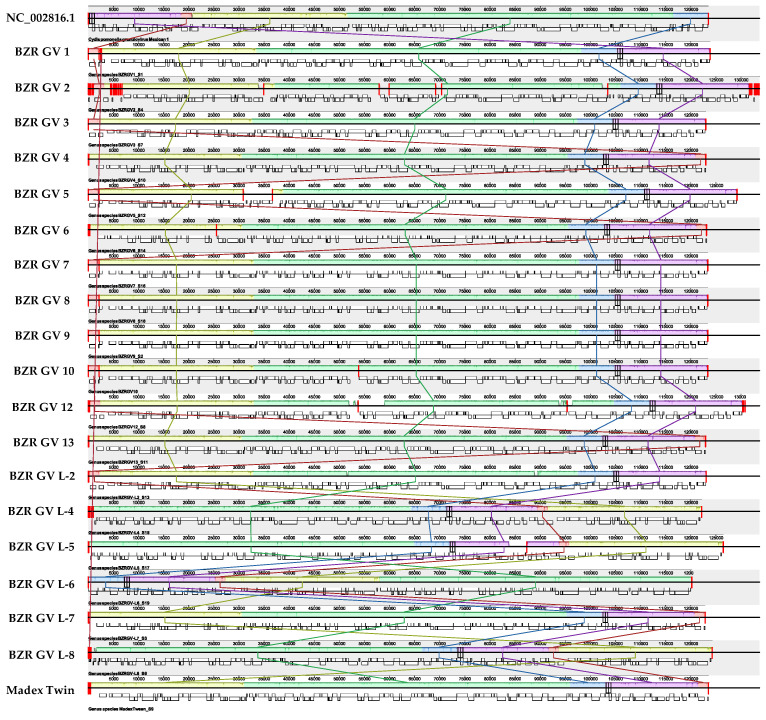
The result of the whole-genome alignment, where colored blocks indicate the height of the similarity profile of the corresponding regions of the genome. The height of the similarity profile corresponds to the average level of conservation in a given genomic sequence region. That is, “dips” in the sequences mean low similarity of this region with respect to the rest of the genome [34]. The first color line shows the reference, from the second to the last—BZR GV 1, BZR GV 2, BZR GV 3, BZR GV 4, BZR GV 5, BZR GV 6, BZR GV 7, BZR GV L-8, BZR GV 9, BZR GV 10, BZR GV 12, BZR GV 13, BZR GV L-2, BZR GV L-4, BZR GV L-5, BZR GV L-6, BZR GV L-7, BZR GV L-8, Madex Twin, respectively. CDS are shown as white boxes under each genome.

Whole-genome alignment was performed using the progressiveMauve program with standard parameters. The obtained results were visualized using the Mauve program [34] (Figure 4).

Whole-genome alignment shows generally close genome similarity, which is consistent with ANI analysis. Large regions with low sequence similarity are shown in the following genomes: BZR GV 2, BZR GV 5, BZR GV 12, BZR GV L-2, BZR GV L-5, and BZR GV L-7. These regions included CDSs with both predicted functions and hypothetical proteins, and CDSs may only partially fall into such regions. We considered regions of 300 bp in size to be large regions.

There appear to be several reasons for this: the effect of fragmented assembly or errors in assembling reads into longer contigs, or accumulated differences contribute to the sequence. Further details are provided for such CDSs in the Supplementary Materials (Table S5). The genes of interest have not fallen within these regions, which is consistent with the analysis of genes and their products involved in the infectious process of virosis.

#### 2.5.3. Gene Repertoire Analysis

The GenAPI tool [35] that allows comparisons of incomplete closely related genomes of microorganisms was used to compare the gene repertoire.

**Figure 5:**
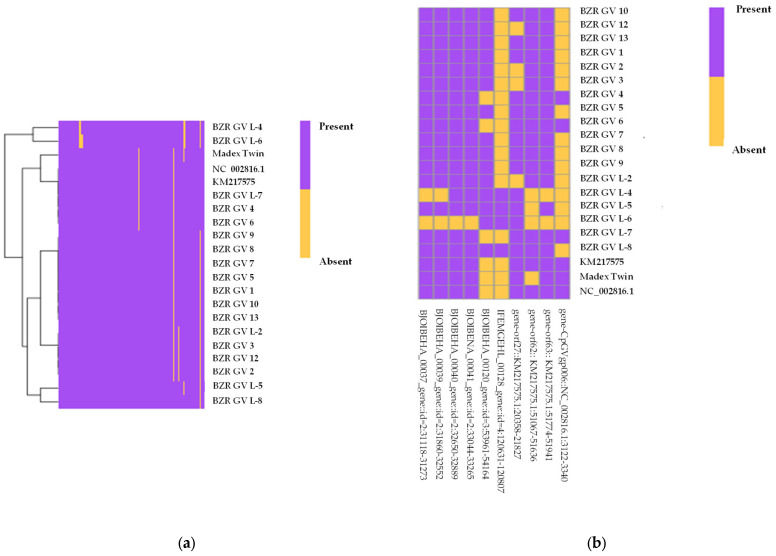
The heatmap of the representation of the gene repertoire of the studied viruses relative to technical and biological references; color indicates gene presence/absence (purple—presence in genomes, yellow—absence in genomes): (**a**) genes across all studied genomes and two references; (**b**) portions of genes across all studied genomes and two references.

Figure 5 shows the result of comparing the gene repertoire of 18 tested CpGV strains, “Madex Twin”, as well as two references: NC_002816.1 and KM217575.

The absence of genes is defined by the similarity thresholds of the compared sequences. Thus, if a gene sequence has a similarity level of less than 90% with less than 50% coverage, the gene is considered absent in this genome relative to the reference.

The names of genes whose presence varied between strains and their products are presented in Table 4, where gene No 1 is listed according to the designations adopted in annotation NC_002816.1, genes No 2–4 are listed according to the designations adopted in annotation KM217575, genes No 5–10 are the genes predicted by the Prokka program in the tested genomes.

Analysis of the gene repertoire allowed us to identify 10 genes present in only a part of the genomes of the studied strains. Among them, there are two genes absent in most of the analyzed strains—orf6 and IFEMGEHL_00128. At the same time, three strains—BZR GV L-7, BZR GV 6, and BZR GV 4—turned out to be the closest in their gene repertoire to “Madex Twin”, which differs from them by the absence of the orf62 gene. Thus, the results obtained are consistent with the results of the comparative genomic study based on the analysis of average nucleotide identity.

#### 2.5.4. Bioinformatic Verification of Detected Deletions

We verified the reports of possible deletions indicated by the GenAPI results by using additional information on changes in the depth of coverage of the corresponding sites (CNVpytor [36]) and their comparison with regions of reduced homology in the results of the whole-genome alignment (progressiveMauve [34]) of the analyzed genomes of *C. pomonella* granulovirus strains. In addition, we compared each such region with the reference gene to find out which genes are affected by the deletion. Additionally, we checked the list of genes from Table 4, whose IDs were predicted by the Prokka program in the studied genomes for consistency with the reference NC_002816.1 by pairwise alignment. It turned out that BJOIBEHA_00037 is orf63 bro, BJOIBEHA_00039 is orf64, BJOIBEHA_00040 is orf65, and BJOIBEHA_00041 is orf66 ptp-2. The designations of these genes as in the reference were used in Table 5.

The analysis confirmed that for BZR GV L-4 and BZR GV L-6, there is a deletion (Table 5) that caused the genomes to lack genes shown by GenAPI. No significant deletions or duplications were identified for the remaining genomes.

Next, we decided to see what processes in the virus these genes are responsible for. However, the functional annotation of genes and their products for CpGV has gaps, some of the genes are labeled as homologous to a gene from another baculovirus. For example, ORF64 is similar to XcGV ORF66, or ORF65 similar to AcMNPV ORF79 [13]. 

Using InterPro [37], we decided to look at the representation of domains in ORF63-ORF66 proteins but only for ORF65 and ORF66 we managed to find domains similar to the known domains in the database. It turned out that ORF65 is characterized by the GIY-YIG endonuclease domain. The literature shows that the GIY-YIG family of nucleases is involved in processes such as mobile element transfer, DNA recombination—including the GIY-YIG domain being associated with DNA repair—and maintenance of genome stability [38,39]. For example, in *Bombyx mori* nuclear polyhedrosis virus (BmNPV), the Bm65 (ORF65) protein is a member of the GIY-YIG nuclease superfamily and is a very important protein that repairs UV-induced damage, and the absence of Bm65 results in a virus phenotype that is more sensitive to UV radiation [40]. Bm65 has been shown to be homologous to ORF79 (Ac 79) of *Autographa californica* multiple nucleopolyhedrovirus (AcMNPV). A study [41] showed that Ac79 (to which ORF65 CpGV is homologous) is required for efficient budded virus production. 

The orf66 is known as a pro-apoptotic protein gene *ptp-2* [13], which corresponds to a protein-tyrosine phosphatase-like domain defined in InterPro that may be involved in post-translational modification. In BmNPV, the *ptp-2* gene is known to increase viral transmission [42].

We also found information in the literature that orf63 bro is homologous to ORFs of the baculovirus repeat (bro). The bro genes represent a multigenic family in baculoviruses and are thought to have an important function in gene transcription and genome replication [13]. In [43], the orf64 gene was identified as homologous to ORFs crle59 and phop56, the functions of which have not been reported.

#### 2.5.5. Analysis of the Gene *pe38* CpGV

The *pe38* gene has previously been shown to be essential for CpGV infectivity and to be a key factor in overcoming resistance to CpGV in the codling moth [44]. Gebhardt et al. found a mutation that represents a 24-nucleotide repeat in *pe38*, which leads to a mutation in the protein in the form of an 8-amino-acid repeat. This repeat is present in the CpGv-M reference genome and its presence is associated with type I resistance. We decided to analyze *pe38* for the presence of such repeats in our genomes.

**Figure 6:**
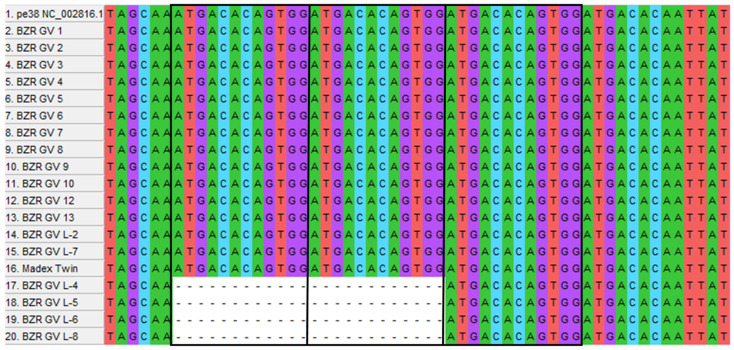
Part of a multiple alignment related to the repeat in the *pe38* gene that results in a DTVD repeat in the PE-38 protein. The nucleotide sequence is shown, with the repeats highlighted in black rectangles. For the BZR GV L-4, BZR GV L-5, BZR GV L-6, and BZR GV L-8 genomes, the absence of repeats is shown with dashed lines on a white background. Multiple alignments are visualized in MEGA 11 [45].

From the 18 genomes of BZR GV and “Madex Twin”, as well as from the reference genome with this insertion, we selected *pe38* gene sequences and performed multiple MAFFT alignment. The alignment showed for the BZR GV genomes L-4, L-5, L-6, and L-8, there was an absence of this repeat. In all other genomes, the repeat is present in the same size as in the reference (Figure 6).

Additionally, for BZR GV L-4, L-5, L-6, and L-8 genomes, the AGCAGCAGCAGTTCGAGCAGCAGGAGA insertion and four SNPs relative to the reference are present in *pe38*.

### 2.6. Classification of BZR GV L-4, BZR GV L-5, BZR GV L-6, and BZR GV L-8 into Genomic Groups

Previously, a description of the genetic diversity of CpGV has been proposed in the literature as a division into genomes of four types, A, B, C, and D, with the C genome being the ancestral form and characterized by the absence of the later acquired orf63-orf66 genes observed in the A, B, and D genomes [46]. More recent articles have proposed a division into seven groups—A, B, C, D, E, F, and G—and three types of resistance [47,48,49]. CpGV strains belonging to genotype C are also known to have reduced virulence against viruses compared with genomes of other types [46].

**Figure 7:**
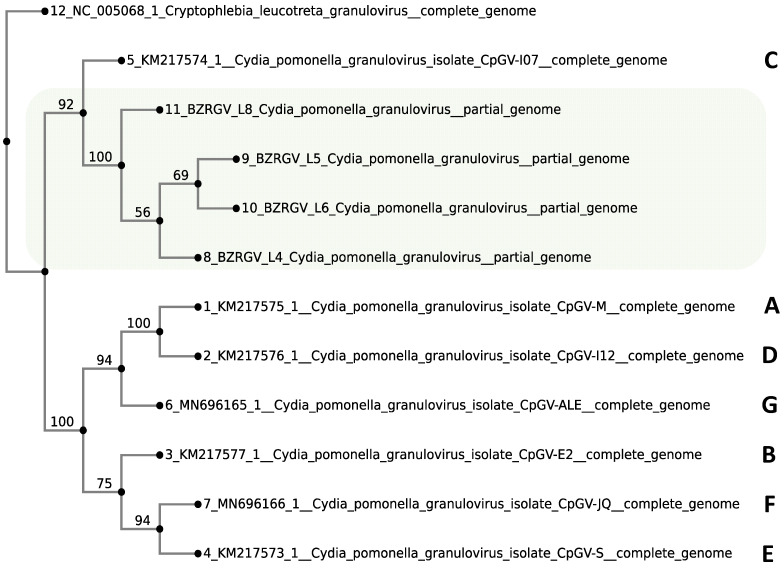
Phylogenetic analysis of twelve CpGV isolates based on nucleotide sequence alignment of whole genomes. The genomic sequence of CrleGV was selected as an outgroup. A total of 500 bootstrap replicates were used. The Jukes–Cantor substitution model was chosen. CpGV genome groups A–G are shown on the right. BZR GV genomes are highlighted in light green color.

We decided to look at how genomes with complete orf63-orf66 genes or with no repeats in the *pe-38* gene are arranged by genomic groups. Phylogenetic analysis is based on complete assemblies of CpGV-M, -I12, -S, -E2, -I07, -ALE, and -JQ genomes, which have already been assigned to the genomic groups A, B, C, D, E, F, and G, and fragmented assemblies of BZR GV L-4, BZR GV L-5, BZR GV L-6, and BZR GV L-8. The complete *Cryptophlebia leucotreta* granulovirus (CrleGV) assembly has been used as an outgroup. Figure 7 shows the phylogenetic tree constructed in the MAFFT web service [32] and visualized in Phylo.io [50]. The genomes BZR GV L-4, BZR GV L-5, BZR GV L-6, and BZR GV L-8 are clustered together with the Iranian isolate CpGV-I07, which is of genotype C. CpGV-I07 has also been shown to lack the orf63-orf66 genes in paper [46].

## 3. Discussion

This paper presents the results of genome sequencing, assembly, and comparative genomic analysis of 16 CpGV strains endemic to southern Russia and two strains from Kazakhstan, as well as the strain included in the commercial preparation “Madex Twin”. The genes and protein products involved in the infectious process of virosis have been compared, and synonymous and missense substitution variants have been identified. Average nucleotide identity (ANI) has demonstrated high similarity with other granulovirus genomes of different geographical origins. The analysis of gene repertoire variation has shown that the BZR GV 4, BZR GV 6, and BZR GV L-7 strains are the closest in their gene repertoire to the strain of the commercial formulation “Madex Twin”. Based on the genotype structure of the BZR GV 4, BZR GV 6, and BZR GV L-7 strains and the reference strain, it can be assumed that they will have similar efficacy against the target insect and mechanism of action, which makes them the most promising microbial biocontrol agents [51]. Nevertheless, in the situation where it is known that the population of *C. pomonella* population is known to have developed resistance to CpGV of genomic group A (in particular, to CpGV-M-based preparations), the strains BZR GV L4, BZR GV L5, BZR GV L6, and BZR GV L8 may be considered as promising, as they carry only one copy of the tandem repeat with the ATGACACAGAGTGG motif in the *pe38* gene (as opposed to the three copies characteristic of CpGV-M), which is the signature of CpGV isolates for breaking type I resistance [44,48]. 

In our study, we have found a viral strain possibly belonging to genotype C (BZR GV L-6), as the orf63-orf66 set is also missing (presumably, genes contained in these loci do not affect virus pathogenicity). However, additional studies are needed to accurately determine whether this genome belongs to genotype C. In terms of the absence of orf63-orf66, the strain of greatest interest is the BZR GV L-4 strain, which could possibly represent a transitional variant between the known C and the rest (A, B, D, E, F, and G) genotypes. While the orf63 and orf64 genes are absent in it, as in the ancestral form, the orf65 and orf66 genes are present, suggesting that the genotype of this strain may be considered as an intermediate in the evolution of CpGV genome types. It should be emphasized that the coevolution of baculovirus strains and host insect immunity is a constant and continuous process that is closely related to the habitat of the latter [52]. Consideration of the frequencies of genotype-specific SNPs and their correlation to resistance types will allow us to identify the most promising virus strains for further study.

It is known from the literature that the use of baculoviruses is possible not only against the target insect but also against closely related species [53,54]. Therefore, the potential of new, previously unknown CpGV strains may be much broader than that of existing cultures, which emphasizes the importance of working with them once again.

In addition to the written above, for the effective use of baculoviruses in the control of phytophagous insects, studies of their compatibility with other bioagents, such as *Bacillus thuringiensis* or *Beauveria bassiana* are necessary [55,56]. The combination of insect viruses with chemical pesticides is also important for creating effective integrated defense systems [57].

It should be noted that the use of baculovirus strains is widespread not only in biomethodology but also in medical research. Thus, to obtain proteins of different origins, a vector baculovirus system is used, where an insect host cell acts as a bioreactor. In this safe and effective way, it is possible to obtain the necessary amount of target protein, which will not differ from the natural analog in its properties [58]. In addition, the baculovirus expression system can serve as an additional component of the one-step cloning system necessary for the creation of multigenic expression constructs [59]. A wide range of proteins, including glycoproteins, recombinant viruses, and vaccines obtained in this way can be used for COVID-19 research, which is especially important in the current world situation [60]. Therefore, new cultures of baculovirus strains should be comprehensively studied to unlock their potential in the future.

Thus, this study demonstrates the insecticidal potential of 18 CpGV strains as a basis for the development of bioinsecticides against *C. pomonella*. The identified similarities/differences in the genetic sequences of the studied samples may be significant in terms of their entomopathogenic activity against the target insect, which requires additional laboratory bioassays using insect populations as well as field trials. It is also necessary to point out some limitations of our study. Although the CpGV genome was sequenced and assembled quite a long time ago [13], the functions of specific genes are still poorly characterized. This includes genes of key importance for the pathogenesis of granulovirus infection and the determination of entomopathogenic properties of specific strains based on the information contained in their genomes. Therefore, in this study, we focused on a number of genes for which there is reliable information on their involvement in pathogenesis, namely genes encoding apoptosis inhibitor (IAP), matrix metalloprotease (MMP), and Chitinase and Cathepsin enzymes. The whole-genome comparisons presented in this work provide a broader picture but do not allow us to judge the underlying mechanisms determining entomopathogenic properties, which require further experimental validation. Further directions of research may be related both to the analysis of the biological efficacy of the presented strains and to the detailed study of the functions of specific CpGV proteins, which are currently poorly characterized. Obtaining new information about this and the integration of knowledge about the course of apple moth granulosis will allow the development of a more systematic approach to the task of biocontrol of this agricultural pest.

## 4. Materials and Methods

### 4.1. Virus Samples

Virus samples were obtained from infected *Cydia pomonella* caterpillars. The caterpillars were collected between 2019 and 2022 in the territories of Kazakhstan and Russia (Krasnodar Krai and Rostov Oblast). The virus strains have shown entomopathogenic properties against the natural population of *C. pomonella* and laboratory population of *Galleria mellonella* [61,62].

Each virus sample was stored as an aqueous suspension or wettable powder in the bioresource collection of the Federal Research Center of Biological Plant Protection’s “State Collection of Entomoacariphages and Microorganisms”. In this research, we used the scientific equipment «Technological line for obtaining microbiological plant protection products of a new generation» (https://ckp-rf.ru/catalog/usu/671367/) (accessed on 30 April 2024).

CpGV strains were developed in vitro using *G. mellonella* by surface inoculation of the diet. Aqueous virus suspensions were obtained by homogenization of infected biomass followed by filtration, centrifugation (Eppendorf 5810 R) at 3000 rpm for 15 min, and resuspension [63,64]. Virus strains were transported in sealed plastic 50 mL tubes.

### 4.2. DNA Extraction and Sequencing

During the molecular–genetic identification of the CpGV strains under study, total DNA was isolated using the PureLink™ Genomic DNA Mini Kit (Invitrogen, Carlsbad, CA, USA). Ultra-sonic DNA fragmentation was carried out on a Covaris M220 device (Covaris, Woburn, MA, USA) with parameters optimized to obtain a maximum of 300 bp fragments (microTUBE 50 AFA; Duty Factor 10; Peak Power 75 W; Cycles/Burst 200; Duration 90 s). The resulting fragments were purified by adding 1.6 volume of Agencourt AMPure XP (Beckman Coulter, Brea, CA, USA). 

Genome library preparation was carried out with 100 mkg of the fragmented DNA using a KAPA Hyper Prep Kit and KAPA Unique Dual-Indexed Adapter Kit according to the manufacturer’s instructions for barcoded libraries. Amplification of libraries was carried out during 9 PCR cycles. The quality and molarity of the resulting libraries were determined using a Bioanalyzer BA2100 with a High Sensitivity DNA Kit (Agilent, Santa Clara, CA, USA). For application, a solution of 18 libraries was prepared in equimolar concentration, the final concentration was 4 nMol. Sequencing of the obtained libraries was carried out on a NextSeq550 device using the NextSeq 550 Mid Output v2 Kit 300 cycles (Illumina, San Diego, CA, USA), with paired-end reads of 2 × 150 bp according to the manufacturer’s protocol with an estimated sequencing volume of 20 million reads per sample.

### 4.3. Genome Assembly and Annotation

Genome sequencing results were quality checked using the FastQC (v. 0.11.9) program [65] with default settings. Overall, the data quality was satisfactory. Trimming of reads was carried out using the fastp (v. 0.22.0) program [66]. We settled on the following parameters for viral samples: --trim_poly_g, -x, -l 15, -p, -D, and -h, where --trim_poly_g is the minimum length to detect polyG at the end of the read; -x is to enable polyX trimming at the 3’ ends; -l is the option to discard reads with lengths less than 15; -p is to analyze overrepresented sequences; -D is to enable deduplication to exclude duplicated reads/pairs; and -h is to output a report of the work in html format. Trimming of reads allowed overrepresented sequences to be excluded from consideration. In most samples, two GC-composition peaks were present each. We hypothesized that this effect could be due to the presence of host insect DNA.

We filtered for possible contamination of reads with host *(Cydia pomonella*) sequences by mapping reads to the virus reference genome (NC_002816.1) using the BWA program (v. 0.7.17) [67] with the default settings for paired reeds. Reads mapped to the reference virus genome were selected using Samtools (v. 1.9) [68,69] without considering the quality of mapQ mapping. We proceeded from the assumption that viruses are rapidly variable and the mapQ parameter can be disregarded.

FastQC quality check was performed again. GC-composition of reads corresponded to the theoretical one. Thus, we were convinced of our assumption about the presence of non-viral DNA in the data.

The NC_002816.1 genome [13] from the NCBI RefSeq database was used as a reference genome of the virus. The genome is represented by a ring chromosome and has a size of 123.5 Kb.

Draft assemblies were performed by two assemblers based on de Bruijn graph construction: Spades (v. 3.13.0) [19,20] for de novo assembly and MinYS (v. 1.1) [21] for reference-guided assembly. Spades was used with default settings. Additionally, the following parameters were specified when assembling MinYS virus genomes: -assembly-kmer-size 41, -assembly-abundance-min 4, -min-contig-size 400, and -nb-cores 8, where -assembly-kmer-size refers to the k-mer size used for Minia assembly (built into MinYS), -assembly-abundance-min is the minimum number of k-mer used for assembly, and -min-contig-size is the minimum counting size was used for gap-filling. These numerical parameters were taken from the information provided by the program developers on github (https://github.com/cguyomar/MinYS/blob/master/doc/tutorial.ipynb accessed on 30 April 2024). Other parameters had default values. The Pilon software tool (v. 1.24) [22] was used to reduce the number of erroneous single-nucleotide substitutions and contig gaps for each assembly. The final assembly was assembled using the Gfinisher tool (v. 1.4) [24] with default settings. The primary assembly was the Spades assembly and the secondary assembly was the MinYS assembly. Each of the assemblies was evaluated for quality with the Quast tool (v. 5.2.0) [23].

Genome annotation was performed using Prokka (v. 1.14.6) [25] with standard parameters. The --kingdom parameter responsible for annotation selection was specified as “viruses”. Blastp and blastn from the Blast+ package (version 2.5.0) [70] were used to identify proteins involved in the infection process of virosis and then the genes synthesizing them. The sequences of MMP, IAP, Chitinase, and Cathepsin proteins were downloaded from the UniprotDB database [26]. Multiple alignment was performed using the web version of MAFFT (v. 7) [32] with default settings.

### 4.4. Comparative Genomic Analysis

For comparative genomic analysis of the studied virus strains, 13 genomes of different CpGV strains of the complete assembly were obtained from the NCBI Virus database (identifiers are given in the Supplementary Materials (Table S6)). Comparison of the obtained genomes with complete virus genome assemblies was performed using FastANI (v. 1.1) [33] to calculate the average nucleotide identity (ANI) across the entire genome without alignment. FastANI was run with the following parameters: --ql, --rl, and --minFrag 35 -output, where -ql is responsible for the list of submitted genomes to be compared, --rl is responsible for the list of genomes we want to compare, and --minFrag means the number of fragments the genome will be split into—the default value is 50. There is also a fragLen parameter, which is responsible for the fragment length (default is 3000). For granulosis viruses, these two parameters were adjusted because the total length of genome sequences is not sufficient for the default parameters. In this case, we left the fragment lengths but reduced their number. The output parameter recorded a text file with genome similarity values. The similarity of genomes was visualized using the gplots library of the R programming language. 

The progressiveMauve (v. 2.4.1) [34] whole-genome alignment tool was used to compare with the reference genome for the presence of mutations at the chromosome level and to identify sites of reduced homology. It was used with default settings. GenAPI (v. 1.0) [35] was used with parameters—-p 4, --tree, and –matrix—to compare patterns of gene presence and absence in viral genomes relative to two CpGV granulovirus reference genomes (NC_002816.1 and KM217575). The tool is remarkable in that it can be used even when only fragmented genome assemblies are available. Based on the information about the depth of coverage of the reference genome by reads, CNV analysis was performed using CNVpytor (v. 1.3.1) [36]. In order to use CNVpytor for the CpGV genome (the human genome is the default: hg19 (GRCh37) and hg38 (GRCh38)), we created a GC and mask file following the example of developers on github (https://github.com/abyzovlab/CNVpytor/blob/master/examples/AddReferenceGenome.md accessed on 30 April 2024). After that, we obtained the imported read depth signal from the BAM files obtained by mapping reads to the reference virus genome and predicted CNV regions as described in the developer’s guide on github. We used CNVpytor to confirm the absence of genes listed in GenAPI that arose due to deletions.

For genes that were confirmed by CNVpytor as missing, domains were searched in web-service InterPro [37] to somehow characterize the proteins for possible functions in CpGV.

### 4.5. Phylogenetic Analysis

To construct the phylogenetic tree, we used complete assemblies of the CpGV-M, -I12, -S, -E2, -I07, -ALE, and -JQ genomes from NCBI Virus (accession numbers are presented in Table S6 of the Supplementary Materials). We also used fragmented genome assemblies of BZR GV L-4, BZR GV L-5, BZR GV L-6, and BZR GV L-8 strains (accession numbers are provided in Table S3 of the Supplementary Materials). Multiple alignments were also performed in the web-service MAFFT (v. 7). For sequence alignment, strains BZR GV L-4, BZR GV L-5, BZR GV L-6, and BZR GV L-8 were reorganized relative to the first ORF in CpGV-M. Genome of the *Cryptophlebia leucotreta* granulovirus (CrleGV) (NC_005068.1) was used as an outgroup.

The phylogenetic tree was constructed using the Neighbor joining algorithm with the Jukes–Cantor substitution model in the web-service MAFFT (v. 7). Bootstrap support was used. The tree was visualized in the Phylo.io service [50].

The main methods used in this study were described above and the data flow diagram is summarized in the schematic that can be found in the Supplementary Materials (Figure S1).

## Figures and Tables

**Table 1:** Characterization of the final CpGV genome assemblies resulting from improvement of the Spades and MinYS draft assemblies using Pilon and GFinisher tools.

Strain	Total Length (bp)	Largest Contig (bp)	Number of Contigs	GC Content (%)	Genome Fraction (%)	Duplication Ratio	Misassemblies	Mismatches	Indels	Indels Length, (bp)
NC_002816.1	123,500	123,500	1	45.27	100	1	0	0	0	0
BZR GV 1	123,848	121,196	4	45.23	99.795	0.999	0	123	83	495
BZR GV 2	133,678	33,116	28	45.25	99.498	1.047	0	204	87	588
BZR GV 3	122,997	122,997	1	45.23	99.499	0.999	0	126	83	495
BZR GV 4	123,038	122,952	2	45.25	99.517	1	0	82	78	212
BZR GV 5	129,218	92,474	4	45.06	99.794	1.046	0	168	102	635
BZR GV 6	123,191	97,631	6	45.26	99.499	1	0	18	68	168
BZR GV 7	123,363	121,196	2	45.23	99.795	0.999	0	124	83	495
BZR GV 8	123,369	121,195	2	45.23	99.795	0.999	0	124	83	495
BZR GV 9	123,364	121,196	2	45.23	99.795	0.999	0	124	83	495
BZR GV 10	123,362	69,475	3	45.23	99.794	0.999	0	124	83	495
BZR GV 12	122,999	122,999	4	45.23	99.499	0.999	0	137	84	496
BZR GV 13	122,929	122,843	2	45.24	99.517	0.999	0	118	81	457
BZR GV L-2	123,126	123,004	2	45.23	99.499	0.999	0	126	83	495
BZR GV L-4	122,210	121,216	4	45.42	98.572	0.993	0	503	169	1268
BZR GV L-5	126,442	87,241	3	45.29	99.792	1.025	0	525	171	1284
BZR GV L-6	120,194	120,194	1	45.44	97.805	0.993	1	503	167	1266
BZR GV L-7	122,862	122,776	2	45.25	99.517	0.998	0	95	79	388
BZR GV L-8	124,273	123,670	4	45.24	99.792	1.001	0	502	168	1281
Madex Twin	123,542	123,071	4	45.27	99.691	1	0	15	67	166

**Table 2:** Characteristics of genome annotations. The ‘predicted proteins’ column indicates the fraction of predicted proteins, with the remainder annotated as ‘hypothetical proteins’.

Strain	Total Length of Assembly (bp)	CDS	Predicted Proteins (%)
BZR GV 1	123,848	138	23.9
BZR GV 2	133,678	148	23
BZR GV 3	122,997	136	24.3
BZR GV 4	123,038	133	24.8
BZR GV 5	129,218	145	24.1
BZR GV 6	123,191	131	25.2
BZR GV 7	123,363	137	24.1
BZR GV 8	123,369	137	24.1
BZR GV 9	123,364	137	24.1
BZR GV 10	123,362	137	24.1
BZR GV 12	130,804	141	24.8
BZR GV 13	122,929	136	24.3
BZR GV L-2	123,126	136	24.3
BZR GV L-4	122,210	128	25
BZR GV L-5	126,442	134	24.6
BZR GV L-6	120,194	125	26.4
BZR GV L-7	122,862	135	24.4
BZR GV L-8	124,273	134	24.6
NC_002816.1	123,500	143	

**Table 3:** Information on NC_002816.1 proteins involved in the infection process, where ID is the protein identifier in the UniprotDB database [26].

ID	Protein Name	Length (aa)	Function	Link to the Study
P41436	IAP	275	apoptosis inhibitor, involved in the realization of cell apoptosis	[27,28]
Q91F09	MMP (matrix metalloprotease)	545	family of zinc-dependent endopeptidases that degrade extracellular matrix proteins	[29]
O91466	Сhitinase	594	an enzyme that causes the breakdown of the insect’s chitinous covering	[30,31]
O91465	Cathepsin	333	a protein involved in the degradation of internal larval tissues	[31]

**Table 4:** The list of genes whose presence differed between the virus strains tested and their products.

No	Gene Name	Product Name	Corresponding Gene Identifiers in Figure 5b
1	*orf6*	ORF6	gene-CpGVgp006::NC_002816.1:3122-3340
2	*orf63*	ORF63	gene-orf63:: KM217575.1:51774-51941
3	*orf62*	ORF62	gene-orf62:: KM217575.1:51067-51636
4	*orf27*	ORF27	gene-orf27::KM217575.1:20358-21827
5	*IFEMGEHL_00128*	hypothetical protein	IFEMGEHL_00128_gene::id=4:120631-120807
6	*BJOIBEHA_00120*	hypothetical protein	BJOIBEHA_00120_gene::id=3:53961-54164
7	*BJOIBEHA_00041*	hypothetical protein	BJOIBENA_00041_gene::id=2:33044-33265
8	*BJOIBEHA_00040*	hypothetical protein	BJOIBEHA_00040_gene::id=2:32650-32889
9	*BJOIBEHA_00039*	hypothetical protein	BJOIBEHA_00039_gene::id=2:31860-32552
10	*BJOIBEHA_00037*	hypothetical protein	BJOIBEHA_00037_gene::id=2:31118-31273

**Table 5:** Comparative analysis for missing genes due to deletions. “+” sign means that a gene is absent, “–“ sign means that a gene is present (according to GenAPI and Mauve analyses).

Strain	CNV Type	CNV Region	CNV Size	List of Genes in the CNV Region	GenAPI Absence	Mauve Absence
BZR GV L-4	deletion	NC_002816.1:51701-54100	2400	orf63 bro	+	+
				orf64	+	+
				orf65	−	−
				orf66	−	−
BZR GV L-6	deletion	NC_002816.1:51701-54100	2400	orf63 bro	+	+
				orf64	+	+
				orf65	+	+
				orf66	+	+

## Data Availability

Genome assemblies and annotations have been submitted at NCBI. The accession numbers are listed in the Supplementary Materials (Table S3).

## Supplementary Materials

The following supporting information can be downloaded at: https://www.mdpi.com/article/10.3390/ijms25137146/s1.
