# Supplementary material for: Assembly and Genome Annotation of Different Strains of Apple Fruit Moth Virus (Cydia pomonella granulovirus)
Source: Int J Mol Sci. 2024 Jun 28;25(13):7146. doi: 10.3390/ijms25137146 (PMC11240899; doi:10.3390/ijms25137146)
Supplement: Supplementary file 1 [file ijms-25-07146-s001.zip › Supplementary Table S1.pdf]

Supplementary Table S1. Characteristics of the obtained genomic libraries for 18 strains of *Cydia pomonella* granulovirus and the genome of the isolate producing bioinsecticide "Madex Twin"

№	Strain	Source DNA concentration ng/µL	Fragmented DNA concentration	Concentration of prepared libraries, ng/µL	Molarity of libraries, nMol	Barcode (KAPA UD)
1	BZR GV 1	46,1	17,2	3,68	14,72	UDI49
2	BZR GV 2	4,35	1,5	1,71	6,84	UDI50
3	BZR GV 3	64,9	26,6	4,3	18,06	UDI51
4	BZR GV 4	87,5	29	2,24	9,41	UDI52
5	BZR GV 5	51,2	12,2	1,75	7,35	UDI53
6	BZR GV 6	134	41	2,14	8,99	UDI54
7	BZR GV 7	53,6	17,8	2,4	10,08	UDI55
8	BZR GV 8	125,4	33,6	4,09	17,18	UDI56
9	BZR GV 9	103,2	38	3	12,60	UDI57
10	BZR GV 10	66,7	12	3,62	15,20	UDI58
11	BZR GV 12	36	8,8	3,44	14,45	UDI59
12	BZR GV 13	99,8	33	3,96	16,63	UDI60
13	BZR GV L-2	52,6	25	2,81	11,80	UDI61
14	BZR GV L-4	24,7	6,8	5	25,00	UDI62
15	BZR GV L-5	74,8	22,4	3,82	16,81	UDI63
16	BZR GV L-6	102,6	26,4	2,47	10,87	UDI64
17	BZR GV L-7	53,8	12,3	2,86	12,01	UDI65
18	BZR GV L-8	51,5	23,5	10	42,00	UDI66
19	Madex Twin	89,6	31,5	7	29,40	UDI67
