# Supplementary material for: Assembly and Genome Annotation of Different Strains of Apple Fruit Moth Virus (Cydia pomonella granulovirus)
Source: Int J Mol Sci. 2024 Jun 28;25(13):7146. doi: 10.3390/ijms25137146 (PMC11240899; doi:10.3390/ijms25137146)
Supplement: Supplementary file 1 [file ijms-25-07146-s001.zip › Supplementary Table S2.pdf]

Supplementary Table S2. Characteristics of reads of the analyzed libraries after mapping to the reference genome of 18 strains of *Cydia pomonella* granulovirus and the genome of the strain producing bioinsecticide "Madex Twin "

№	Stain	Average reading length, bp	Number of mapped readings, pc	Number of non-mapped readings, pc	Coverage rate, x
1	BZR GV 1	147	338707	42003081	402,43
2	BZR GV 2	147	2420222	41662646	2875,54
3	BZR GV 3	147	319438	5045376	379,53
4	BZR GV 4	145	719532	45057522	843,68
5	BZR GV 5	145	449308	43078240	526,59
6	BZR GV 6	146	1454397	42551369	1717,53
7	BZR GV 7	146	216656	45687008	256,66
8	BZR GV 8	142	844743	42961295	969,64
9	BZR GV 9	143	125207	48650601	144,74
10	BZR GV 10	142	603465	45106113	692,84
11	BZR GV 12	143	1330050	47944286	1537,82
12	BZR GV 13	145	457599	45200381	536,31
13	BZR GV L-2	147	371224	43751520	441, 01
14	BZR GV L-4	138	657257	33972431	733,25
15	BZR GV L-5	144	423749	45288401	493,26
16	BZR GV L-6	141	298059	46795285	339,72
17	BZR GV L-7	136	485947	66853493	534,34
18	BZR GV L-8	147	195954	52665374	232,88
19	Madex Twin	145	308415	49413757	361, 31
