# Supplementary material for: Assembly and Genome Annotation of Different Strains of Apple Fruit Moth Virus (Cydia pomonella granulovirus)
Source: Int J Mol Sci. 2024 Jun 28;25(13):7146. doi: 10.3390/ijms25137146 (PMC11240899; doi:10.3390/ijms25137146)
Supplement: Supplementary file 1 [file ijms-25-07146-s001.zip › Supplementary Table S3.pdf]

Supplementary Table S3. Accession numbers of *Cydia pomonella* granulovirus sequences in NCBI obtained by this work

№	Isolate	Id sequences in genome assembly	Accession numbers of sequences in NCBI
1	BZR GV 1	id=1<br>id=2<br>id=3<br>id=4	OR743626<br>OR743627<br>OR743628<br>OR743629
2	BZR GV 2	id=1<br>id=2<br>id=3<br>id=4<br>id=5<br>id=6<br>id=7<br>id=8<br>id=9<br>id=10<br>id=11<br>id=12<br>id=13<br>id=14<br>id=15<br>id=16<br>id=17<br>id=18<br>id=19<br>id=20<br>id=21<br>id=22<br>id=23<br>id=24<br>id=25<br>id=26<br>id=27<br>id=28	OR743630<br>OR743631<br>OR743632<br>OR743633<br>OR743634<br>OR743635<br>OR743636<br>OR743637<br>OR743638<br>OR743639<br>OR743640<br>OR743641<br>OR743642<br>OR743643<br>OR743644<br>OR743645<br>OR743646<br>OR743647<br>OR743648<br>OR743649<br>OR743650<br>OR743651<br>OR743652<br>OR743653<br>OR743654<br>OR743655<br>OR743656<br>OR743657
3	BZR GV 3	id=1	OR743658
4	BZR GV 4	id=1_gv4<br>id=2_gv4	OR743675<br>OR743676
5	BZR GV 5	id=1_gv5<br>id=2_gv5<br>id=3_gv5<br>id=4_gv5	OR743677<br>OR743678<br>OR743679<br>OR743680
6	BZR GV 6	id=1<br>id=2<br>id=3	OR743681<br>OR743682<br>OR743683

		id=4<br>id=5<br>id=6	OR743684<br>OR743685<br>OR743686
7	BZR GV 7	id=1_gv7<br>id=2_gv7	OR743687<br>OR743688
8	BZR GV 8	id=1_gv8<br>id=2_gv8	OR743689<br>OR743690
9	BZR GV 9	id=1_gv9<br>id=2_gv9	OR743691<br>OR743692
10	BZR GV 10	id=1_gv10<br>id=2_gv10<br>id=3_gv10	OR743693<br>OR743694<br>OR743695
11	BZR GV 12	id=1<br>id=2<br>id=3<br>id=4<br>id=5<br>id=6	OR743696<br>OR743697<br>OR743698<br>OR743699<br>OR743700<br>OR743701
12	BZR GV 13	id=1_gv13<br>id=2_gv13	OR743702<br>OR743703
13	BZR GV L-2	Seq1_L2<br>Seq2_L2	OR743659<br>OR743660
14	BZR GV L-4	Seq1_L4<br>Seq2_L4<br>Seq3_L4<br>Seq4_L4	OR743661<br>OR743662<br>OR743663<br>OR743664
15	BZR GV L-5	Seq1_L5<br>Seq2_L5<br>Seq3_L5	OR743665<br>OR743666<br>OR743667
16	BZR GV L-6	Seq1_L6	OR743668
17	BZR GV L-7	Seq1_L7<br>Seq2_L7	OR743669<br>OR743670
18	BZR GV L-8	Seq1_L8<br>Seq2_L8<br>Seq3_L8<br>Seq4_L8	OR743671<br>OR743672<br>OR743673<br>OR743674
