# Supplementary material for: Assembly and Genome Annotation of Different Strains of Apple Fruit Moth Virus (Cydia pomonella granulovirus)
Source: Int J Mol Sci. 2024 Jun 28;25(13):7146. doi: 10.3390/ijms25137146 (PMC11240899; doi:10.3390/ijms25137146)
Supplement: Supplementary file 1 [file ijms-25-07146-s001.zip › Supplementary Table S5.pdf]

Supplementary Table S5. Information on CDS located in large regions with low sequence similarity. Here CDS are labeled as locus_tag, also as in the annotation, "Product" means whether the translated CDS has predicted function, and "CDS coverage" means the low similarity region contains the full CDS or part of it.

Strain	Locus_tag (gene)	Product	CDS coverage
BZR GV 2	NJLGDECI_00001	hypothetical protein	complete
BZR GV 2	NJLGDECI_00004	hypothetical protein	complete
BZR GV 2	NJLGDECI_00005	hypothetical protein	complete
BZR GV 2	NJLGDECI_00039	Per os infectivity factor 0	complete
BZR GV 2	NJLGDECI_00069	putative 38.0 kDa protein in P143-LEF5 intergenic region	complete
BZR GV 2	NJLGDECI_00070	hypothetical protein	complete
BZR GV 2	NJLGDECI_00082	hypothetical protein	complete
BZR GV 2	NJLGDECI_00113	putative DNA-directed RNA polymerase catalytic subunit	partical
BZR GV 2	NJLGDECI_00114	hypothetical protein	partical
BZR GV 2	NJLGDECI_00148	hypothetical protein	complete
BZR GV 5	JPLFIAAL_00035	hypothetical protein	complete
BZR GV 5	JPLFIAAL_00036	hypothetical protein	complete
BZR GV 5	JPLFIAAL_00037	hypothetical protein	complete
BZR GV 5	JPLFIAAL_00038	hypothetical protein	complete
BZR GV 5	JPLFIAAL_00039	hypothetical protein	complete
BZR GV 5	JPLFIAAL_00040	hypothetical protein	complete
BZR GV 5	JPLFIAAL_00041	DNA-directed RNA polymerase subunit p47	complete
BZR GV 5	JPLFIAAL_00042	Protein ADP-ribose pyrophosphatase ORF38	complete
BZR GV 12	ICELHAMJ_00062	hypothetical protein	partical
BZR GV 12	ICELHAMJ_00063	hypothetical protein	complete
BZR GV 12	ICELHAMJ_00064	hypothetical protein	complete
BZR GV 12	ICELHAMJ_00065 (LEF-5-2)	Late expression factor 5	complete
BZR GV 12	ICELHAMJ_00066	putative 38.0 kDa protein in P143-LEF5 intergenic region	complete
BZR GV 12	ICELHAMJ_00067	hypothetical protein	complete
BZR GV 12	ICELHAMJ_00068 (HELI)	DNA replication helicase	partical
BZR GV 12	ICELHAMJ_00104 (AN)	Alcaine nuclease	partical

BZR GV 12	ICELHAMJ_00105	hypothetical protein	partical
BZR GV 12	ICELHAMJ_00106	hypothetical protein	partical
BZR GV L-2	BGFFOGFG_00062	hypothetical protein	partical
BZR GV L-2	BGFFOGFG_00100	hypothetical protein	partical
BZR GV L-5	JEDKAPMP_00091	hypothetical protein	complete
BZR GV L-5	JEDKAPMP_00092	hypothetical protein	complete
BZR GV L-5	JEDKAPMP_00093	hypothetical protein	complete
BZR GV L-5	JEDKAPMP_00094	hypothetical protein	complete
BZR GV L-7	DDAPCIDK_0002	hypothetical protein	complete
BZR GV L-7	DDAPCIDK_0003	hypothetical protein	complete
BZR GV L-7	DDAPCIDK_0004	hypothetical protein	partical
