# Supplementary material for: Assembly and Genome Annotation of Different Strains of Apple Fruit Moth Virus (Cydia pomonella granulovirus)
Source: Int J Mol Sci. 2024 Jun 28;25(13):7146. doi: 10.3390/ijms25137146 (PMC11240899; doi:10.3390/ijms25137146)
Supplement: Supplementary file 1 [file ijms-25-07146-s001.zip › Supplementary Table S6.pdf]

Supplementary Table S6. Identifiers of complete assemblies of the *Cydia pomonella* granulovirus sequence in NCBI Virus

№	Isolate	Accession number	Country of origin
1	CpGV-ALE	MN696165	China
2	CpGV-JQ	MN696166	China
3	CpGV-KS1	MN696167	China
4	CpGV-KS2	MN696168	China
5	CpGV-WW	MN696169	China
6	CpGV-ZY2	MN696170	China
7	CpGV-ZY	MN696171	China
8	CpGV-SA	MN075941	South Africa
9	CpGV-S	KM217573	Canada
10	CpGV-I07	KM217574	Iran
11	CpGV-M	KM217575	Mexico
12	CpGV-I12	KM217576	Iran
13	CpGV-E2	KM217577	England
