# Supplementary figures and images for: Assembly and Genome Annotation of Different Strains of Apple Fruit Moth Virus (Cydia pomonella granulovirus)
Source: Int J Mol Sci. 2024 Jun 28;25(13):7146. doi: 10.3390/ijms25137146 (PMC11240899; doi:10.3390/ijms25137146)

Figure S1: Data flow diagram and the main methods we used in our work

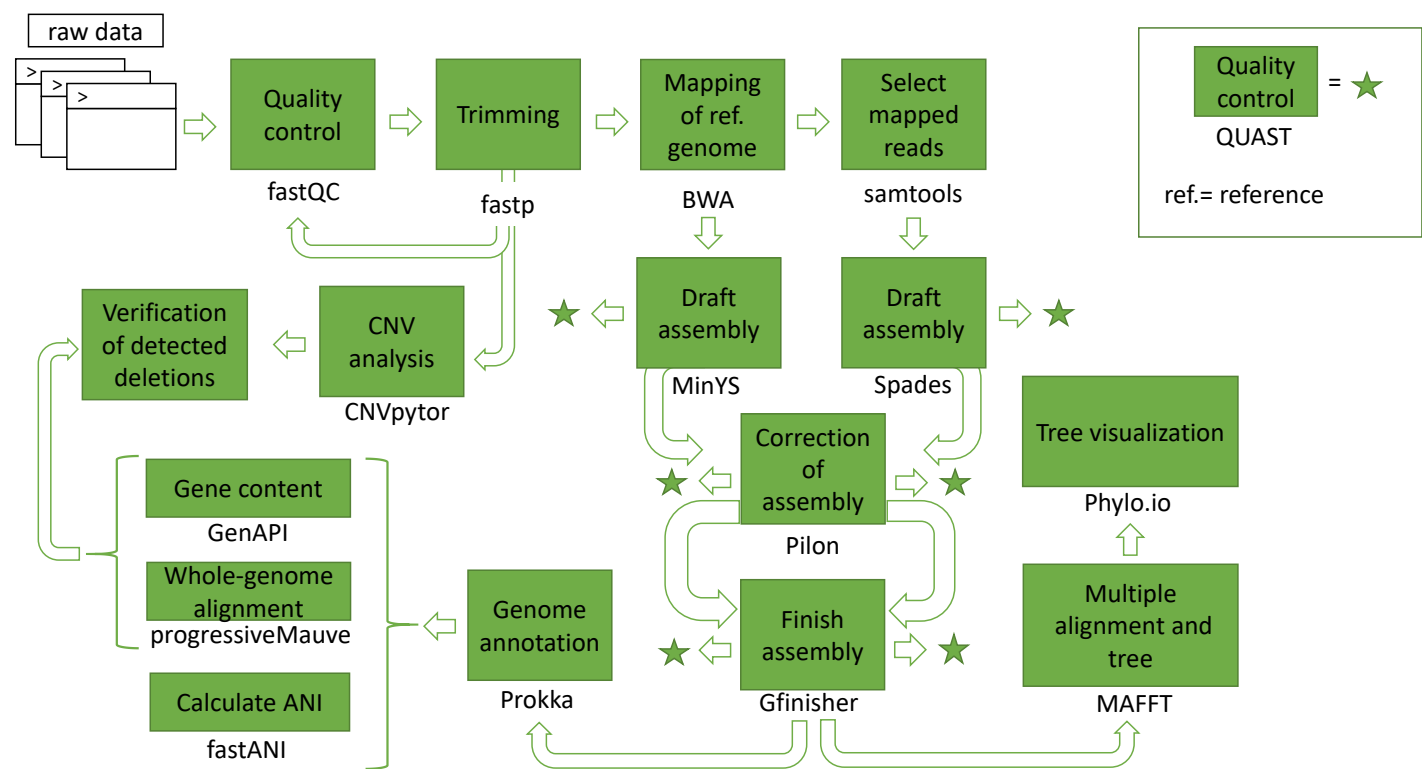

Supplement: Supplementary file 1 [file ijms-25-07146-s001.zip › Figure S1.pdf]
